# Alda-1 attenuates hyperoxia-induced mitochondrial dysfunction in lung vascular endothelial cells

**DOI:** 10.18632/aging.102012

**Published:** 2019-06-17

**Authors:** Sahebgowda Sidramagowda Patil, Helena Hernández-Cuervo, Jutaro Fukumoto, Venkata Ramireddy Narala, Smita Saji, Monica Borra, Matthew Alleyn, Muling Lin, Ramani Soundararajan, Richard Lockey, Narasaiah Kolliputi, Lakshmi Galam

**Affiliations:** 1 University of South Florida, Division of Allergy and Immunology, Department of Internal Medicine, College of Medicine, Tampa, Florida 33612, United States; 2 University of South Florida, Division of Allergy and Immunology, Department of Molecular Medicine, College of Medicine, Tampa, Florida 33612, United States; 3 Department of Zoology, Yogi Vemana University, Kadapa, India

**Keywords:** acute lung injury, hyperoxia, ALDH2, Alda-1, HMVEC

## Abstract

Acute lung injury (ALI) is a major cause of morbidity and mortality worldwide, especially in aged populations. Mitochondrial damage is one of the key features of ALI. Hyperoxia-induced lung injury model in mice has been widely used for ALI study because it features many ALI phenotypes including, but not limited to, mitochondrial and vascular endothelial cell damage. Recently, accumulating evidence has shown that mitochondrial aldehyde dehydrogenase 2 (ALDH2) has a protective effect against oxidative stress mediated cell damage in epithelial cells. However, it is not known whether ALDH2 protects against oxidative stress in vascular endothelial cells. In this current study, we attempted to find the capacity of Alda-1 [(N-(1,3benzodioxol-5-ylmethyl)-2,6- dichloro-benzamide), an ALDH2 activator] to protect against oxidative stress in human microvascular endothelial cells (HMVEC). HMVEC pretreated with Alda-1 prior to hyperoxic exposure vs non-treated controls showed i) lower 4-hydroxynonenal (4-HNE) levels, ii) significantly decreased expressions of Bax and Cytochrome C, iii) partially restored activity and expression of ALDH2 and iv) significantly improved mitochondrial membrane potential. These results suggest that ALDH2 protein in lung vascular endothelial cells is a promising therapeutic target for the treatment of ALI and that Alda-1 is a potential treatment option.

## Introduction

Aging is a major risk factor that contributes to the initiation and development of numerous acute and chronic lung diseases [1]. Aging is related with physiological decline in lung function and, accordingly, mortality due to lung disease with age. Acute lung injury (ALI) is a severe respiratory condition that affects over 200,000 people in the United States annually with an increased incidence in aged populations [1–3]. The most severe form of ALI is Acute Respiratory Distress Syndrome (ARDS). Both the incidence of ALI/ARDS and subsequent mortality rates for patients with ALI/ARDS are increasing, totaling 70,000 deaths annually in the US alone [4]. Oxygen therapy is the most widely used intervention to counteract hypoxemia in ALI/ARDS treatment [5]. However, *in vivo* and *in vitro* studies have indicated that extended exposure to highly concentrated oxygen can induce severe and lethal oxidative damage to the lung [6]. The cellular and molecular mechanism regarding how highly concentrated oxygen causes lung damage is well established. Both epithelial and endothelial cells of the lung are among the major target cells that are severely damaged by oxygen toxicity [7,8]. Endogenously generated reactive oxygen species (ROS) under hyperoxic conditions cause dysfunction and apoptosis of these cell types, which in turn accelerate inflammatory responses such as immune cell recruitment and secretion of inflammatory cytokines [9,10]. While the clinical relevance of lung epithelial cell damage to ALI/ARDS has been extensively studied, the involvement of endothelial cells in these conditions remains unclear. Especially, it is unclear how lung vascular endothelial cells restore their functional integrity under recovery phase after hyperoxic conditions.

Currently, there are various animal models available to study ALI/ARDS; among which, hyperoxia-induced lung injury model has been most widely used as a feasible tool to directly induce elevation of oxidants in the lung [11]. Long-term exposure to hyperoxia causes buildup of ROS and decreases cell viability [6]. Furthermore, hyperoxia-induced lung injury model involves a negligible amount of lung fibrosis when compared to other lung injury models [4,12]. Thus, it is well established that there are many features shared between hyperoxia-induced lung injury model and ALI/ARDS [13]. Hyperoxic insult to the lung generates oxidative stress, which leads to the accumulation of 4-hydroxynonenal (4-HNE), a toxic aldehyde. 4-HNE is an alpha beta unsaturated aldehyde formed by lipid peroxidation. 4-HNE causes mitochondrial damage and cell transduction impairment, as previously reported [13–15]. 4-HNE also causes harmful effects on a variety of tissues and cell types [14,16–19]. ALDH2 has been demonstrated to be a promising target because it metabolizes and counteracts 4-HNE [20]. Therefore, it is reasonable to investigate whether activation of ALDH2 attenuates hyperoxia-induced mitochondrial damage and cell death in lung epithelial and endothelial cells.

Alda-1, a most widely used ALDH2 agonist, reduces the Km value (Michaelis constant) and increases the V-max (maximum velocity) of the acetaldehyde metabolizing reaction catalyzed by ALDH2 [20]. Studies have demonstrated that Alda-1 binds to ALDH2 near the Glu286 and Cys302 residues and thereby enhances the catalytic activity [20–22]. Preclinical effects of Alda-1 have been evaluated in a variety of cell lines and organs. Administration of Alda-1 is known to i) enhance the ALDH2 function in epithelial cells and human umbilical endothelial cells and ii) reduce cerebral and cardiac ischemia, by reducing toxic 4-HNE levels [16,19,23,24].

Lung vascular endothelial cells play an important role in maintaining the functional integrity of vasculature and hence lung homeostasis. They are also involved in vascular remodeling, angiogenesis, coagulation, and blood circulation [25]. Given their integral roles in lung homeostasis, death of lung vascular endothelial cells and consequent loss of vascular integrity represents a significant contribution to the pathogenesis of oxidative stress-induced lung injury [6]. Experimentally delivered high level oxygen triggers epithelial and endothelial cell damage *in vivo,* leading to disruption of blood-air barrier and ending up in edema of the lung. This is a well-known initial disease progression step of ALI/ARDS [26]. While ALDH2 has been demonstrated to serve as a shield against oxidative stress-mediated cell damage in lung epithelial cells [23], it is not known whether ALDH2 protects against oxidative stress in lung vascular endothelial cells.

In the current study we attempt to determine if the pretreatment with Alda-1 is sufficient to attenuate hyperoxia-induced oxidative damage in lung vascular endothelial cells.

## RESULTS

### ALDH2 activation through Alda-1 treatment attenuates 4-HNE accumulation in pulmonary vascular endothelial cells

**Figure 1:**
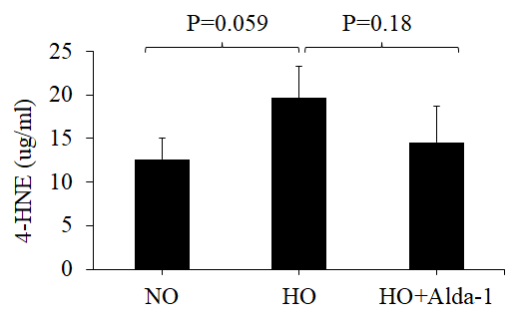
**Activation of ALDH2 diminishes hyperoxia-induced 4-HNE accumulation in lung vascular endothelial cells.** HMVEC were cultured in three different conditions: NO (normoxia), HO (48 hrs of hyperoxia) or HO+Alda-1 (Alda-1 pretreatment followed by 48 hrs of hyperoxia). Whole cell lysates (50 µg protein equivalent) were subjected to ELISA to evaluate the amount of 4-HNE content. The results are shown in mean ± SEM (n=3). Data presented are representative of three independent experiments.

Oxidative stress causes accumulation of 4-HNE, a highly electrophilic aldehyde that is harmful to the cell. ALDH2 catalyzes the conversion of 4-HNE to an inactive form 4-hydroxy-2-nonenoic acid (4-HNA) [27]. Alda-1, an ALDH2 activator has been shown to protect numerous cell types against oxidative stress by increasing ALDH2 activity [16,28–31]. However, no study has verified its protective effect on lung vascular endothelial cells. To verify its anti-oxidative effect on lung endothelial cells, we exposed human microvascular endothelial cells (HMVEC) to hyperoxia in the presence or absence of Alda-1. Hyperoxia causes 55% increase in 4-HNE compared to Normoxia and pretreatment with Alda-1 in hyperoxic conditions causes 26% decrease compared to hyperoxia. The results show that pretreatment with Alda-1 attenuates hyperoxia-induced 4-HNE accumulation in HMVEC (Fig. 1). These results indicate that ALDH2 plays a vital role in protecting lung vascular endothelial cells through the catalytic inactivation of 4-HNE.

### ALDH2 activation through Alda-1 treatment attenuates hyperoxia-induced apoptosis in pulmonary vascular endothelial cells

**Figure 2:**
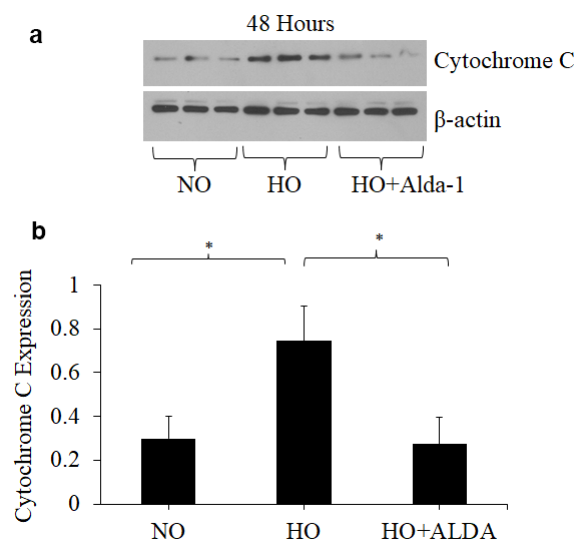
**Activation of ALDH2 attenuates hyperoxia-induced increase in cytochrome c expression in lung vascular endothelial cells.** (**a**) Whole cell lysates extracted from HMVEC cultured under different conditions (normoxia, 48 hrs of hyperoxia, or Alda-1 pretreatment followed by 48 hrs of hyperoxia) were evaluated for cytochrome C levels by Western blotting. Equal amounts of protein (20µg) were loaded per each lane. (**b**) Expression of Cytochrome C was normalized to β-actin and presented in arbitrary units. The results are shown in mean ± SEM (n=3). Data presented are representative of two independent experiments.

**Figure 3:**
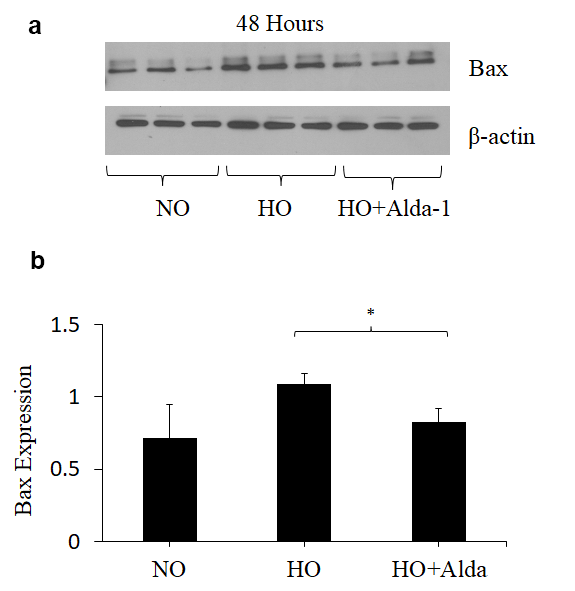
**ALDH2 activation via Alda-1 attenuates hyperoxia-induced increase in Bax expression in lung vascular endothelial cells.** (**a**) Whole cell lysates extracted from HMVEC cultured under different conditions (normoxia, 48 hrs of hyperoxia, or Alda-1 pretreatment followed by 48 hrs of hyperoxia) were evaluated for Bax levels by Western blotting. Equal amounts of protein (20µg) were loaded per each lane. (**b**) Expression of Bax was normalized to β-actin and presented in arbitrary units. The results are shown in mean ± SEM (n=3). Data presented are representative of two independent experiments.

Oxidative stress causes apoptotic and non-apoptotic cell death in many cell types. Cytochrome C and Bax levels reflect apoptotic cell death [14]. To assess whether ALDH2 activation through Alda-1 suppresses apoptosis signaling caused by oxidative stress in HMVEC, the protein levels of Cytochrome C and Bax in whole cell lysate were quantified by Western blotting. The results show that hyperoxia causes 2.5- and 1.5-fold increase in Cytochrome C and Bax levels, respectively, compared to normoxic controls (Fig. 2 and 3). HMVEC treated with Alda-1 prior to hyperoxia resulted in a significant 64% and 25% decrease in the expression of Cytochrome C and Bax, respectively, compared to those untreated with Alda-1 (Fig. 2 and 3). These results indicate that ALDH2 activation protects from oxidative stress-induced apoptosis in lung vascular endothelial cells.

### ALDH2 activation through Alda-1 treatment increases ALDH2 expression in pulmonary endothelial cells during hyperoxia

**Figure 4:**
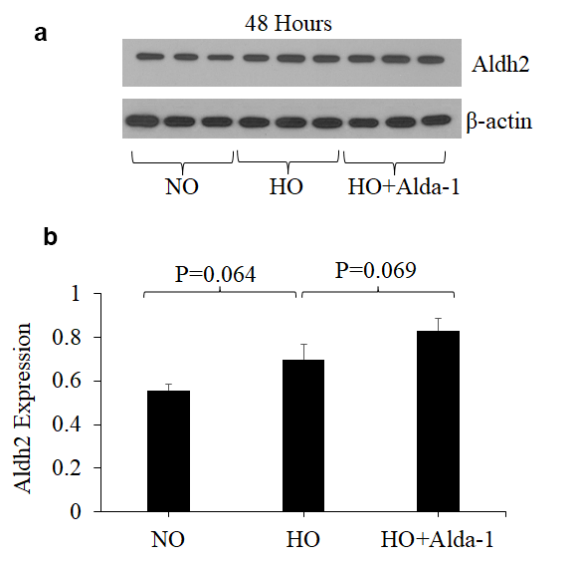
**ALDH2 activation through Alda-1 pretreatment enhances ALDH2 expression in lung vascular endothelial cells under hyperoxia.** (**a**) Whole cell lysates extracted from HMVEC cultured under different conditions (normoxia, 48 hrs of hyperoxia, or Alda-1 pretreatment followed by 48 hrs of hyperoxia) were evaluated for ALDH2 levels by Western blotting. Equal amounts of protein (20µg) were loaded per each lane. (**b**) Expression of ALDH2 was normalized to β-actin and presented in arbitrary units. The results are shown in mean ± SEM (n=3). Data presented are representative of three independent experiments.

**Figure 5:**
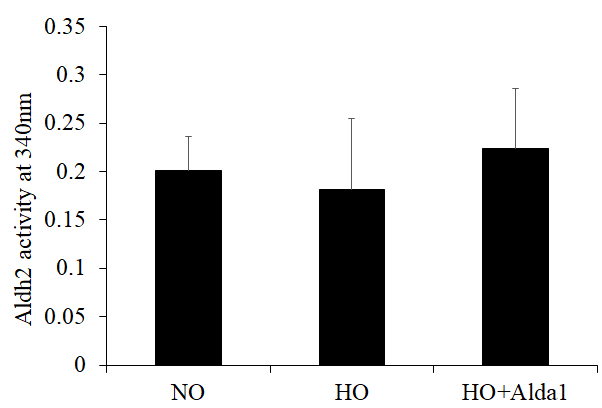
**ALDH2 activation through Alda-1 pretreatment attenuates hyperoxia-induced decrease in ALDH2 activity in lung vascular endothelial cells.** Mitochondrial lysates extracted from HMVEC cultured under different conditions (normoxia, 48 hrs of hyperoxia, or Alda-1 pretreatment followed by 48 hrs of hyperoxia) were subjected to an enzymatic assay to evaluate ALDH2 activity. The results are shown in mean ± SEM (n=3). Data presented are representative of three independent experiments

Recent studies have shown that Alda-1 does not affect the expression levels of ALDH2 but does increase ALDH2 enzymatic activity in various cell types [19,32]. However, no study has examined the effects of Alda-1 on the expression and enzymatic activity of ALDH2 in lung vascular endothelial cells. In order to answer this question, the expression and enzymatic activity of ALDH2 were evaluated. The results show that hyperoxia alone without Alda-1 treatment causes 26% increase in ALDH2 expression in HMVEC though the difference is not significant (p=0.064) (Fig. 4). Cells pretreated with Alda-1 prior to hyperoxia show further increase in ALDH2 expression compared to those untreated with Alda-1 prior to hyperoxia though the difference is not significant (p = 0.069) (Fig. 4). Next, we evaluated the ALDH2 activity. The results show that hyperoxia-treated cells show slight decrease in ALDH2 activity compared to normoxic controls (Fig. 5). Cells pretreated with Alda-1 prior to hyperoxia display 23% increase in ALDH2 activity compared to those untreated with Alda-1 prior to hyperoxia (p=0.48) (Fig. 5). These results indicate that Alda-1 treatment counteract against oxidative stress-induced decrease in the expression and activity of ALDH2 in lung vascular endothelial cells.

### ALDH2 activation through Alda-1 treatment protects pulmonary vascular endothelial cells against hyperoxia-induced mitochondrial damage

Decrease in the mitochondrial membrane potential (membrane depolarization; ΔΨm) represents mitochondrial damage [33]. A fluorescent dye, JC-1, allows us to detect mitochondrial membrane depolarization as a shift in fluorescent signal from red (JC-1 aggregates) to green (JC-1 monomer) and thereby evaluate the extent of the mitochondrial damage of the cells *in vitro*.

**Figure 6:**
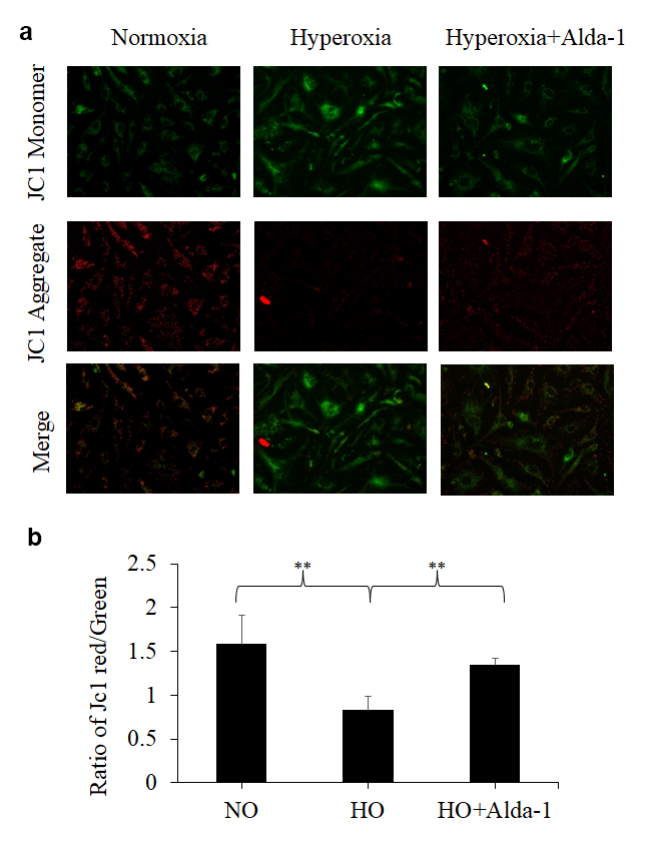
**ALDH2 activation protects lung vascular endothelial cells against hyperoxia-induced mitochondrial membrane damage.** (**a**) HMVEC cultured under different conditions (normoxia, 48 hrs of hyperoxia, or Alda-1 pretreatment followed by 48 hrs of hyperoxia) were subjected to JC-1 staining (magnification=200x). (**b**) Fluorescence intensities of green and red signals were quantified for each image using ImageJ software. The calculated ratios of red to green signals were expressed in arbitrary units. The results are shown in mean ± SEM (n=3). Data presented are representative of three independent experiments.

To evaluate the favorable effect of ALDH2 on mitochondrial function on lung vascular endothelial cells, JC-1 assay was performed on HMVEC cultured under different conditions. As an index for the functional integrity of mitochondria, the ratio of red to green signals was used (referred to as mitochondrial functionality index hereinafter). The results show that HMVEC exposed to hyperoxia vs normoxia controls display 48% significant decrease in mitochondrial functionality index. Cells pretreated with Alda-1 prior to hyperoxia exposure show 62% significant increase in mitochondrial functionality index when compared to the cells untreated with Alda-1 prior to hyperoxia. These results suggest that ALDH2 plays a pivotal role in maintaining the functional integrity of mitochondria in lung vascular endothelial cells (Fig. 6). The results also indicate that ALDH2 activation helps to attenuate oxidative stress-induced mitochondrial damage in lung vascular endothelial cells.

## DISCUSSION

For the first time, it is demonstrated in the current study that ALDH2 activation through Alda-1 treatment has the potential to mitigate hyperoxia-induced functional deterioration of ALDH2 in pulmonary vascular endothelial cells. ALDH2 activation through Alda-1 administration also resulted in significant decrease in Cytochrome C expression and dramatically improved mitochondrial membrane potential. Alda-1 displayed distinguished protection against hyperoxia in HMVEC, as shown by the diminished levels of Bax, an apoptotic activator indicative of mitochondrial stress.

Aging is known to cause significant changes in pulmonary physiology, including alterations of the structure of lungs and the mechanical properties of gas exchange [34]. Almost all the patients suffering from ALI/ARDS receive oxygen therapy to counteract the severe hypoxemia caused by pulmonary edema [35]. However ample evidence demonstrates that extended exposure to highly concentrated oxygen can cause oxidative stress-induced lung damage. Given that the ability of the lung to maintain its homeostasis wanes gradually with age, older patients are considered to be more susceptible to toxic side effects of oxygen [36]. During hyperoxic exposure, the increased oxidative stress results in intracellular accumulation of ROS, thereby causing cellular damage and apoptosis that culminates in tissue and organ failure [6,37]. Mitochondrial ALDH2 plays a pivotal role in preventing the accumulation of 4-HNE, oxidative stress, apoptosis, and mitochondrial membrane damage [13,19,22,38]. ALDH2 has also been implicated in numerous age-related pathologies, such as ischemic heart disease, Alzheimer’s disease, and Parkinson’s disease. Accordingly, an ALDH2 activator Alda-1 has been shown to reduce injuries in the heart, liver, brain, kidney, and intestines. However, Alda-1 has not been thoroughly tested in the lung [16,28–31]. For the first time, we have demonstrated the ALDH2 activation through Alda-1 attenuates hyperoxia-induced cell damage in lung vascular endothelial cells. We also revealed that this protection by Alda-1 is mainly through suppression of apoptosis and improvement of mitochondrial membrane potential.

Previous studies show that hyperoxic exposure induces the formation of 4-HNE-protein adducts, leading to the functional loss of the adducted proteins [38,39]. Moreover, such protein alterations result in impaired cellular feedback, further promoting the production of ROS and oxidative stress and ultimately leading to cellular dysfunction and death [13,32]. Our current study for the first time shows that such ALDH2-assisted cell protection occurs in lung vascular endothelial cells as well.

Exposure of lung cells to hyperoxia for prolonged durations causes a greater decrease in mitochondrial membrane potential mediated through oxidative stress [7,8]. Hyperoxia-induced loss of mitochondrial membrane potential was significantly attenuated by pretreatment with Alda-1 (Figure 6a). The restoration of the expression and activity of ALDH2 and improvement of mitochondrial membrane potential in Alda-1 pretreated lung vascular endothelial cells indicate that ALDH2 is a promising therapeutic target for protecting endothelial cells from oxidative stress-induced cellular damage (Figures 6, 5 & 4, respectively).

Epithelial and endothelial cells are the key components of the blood–air barrier in the lung and its disruption causes increased lung permeability, leading to an influx of fluid from vasculature into the alveoli [26]. Endothelial cells apoptosis is a key initial event that characterizes ALI/ARDS [40]. Therefore, prevention of endothelial cell apoptosis is a reasonable therapeutic intervention for the treatment of ALI/ARDS. In the current study, we show for the first time that ALDH2 activation through Alda-1 mitigates hyperoxia-induced increase in apoptosis-related proteins, Bax and Cytochrome C, in lung vascular endothelial cells. Since hyperoxia-induced accumulation of 4-HNE as well as the mitochondrial membrane potential abnormality are also restored by Alda-1 pretreatment, we surmise that the ALDH2 protects endothelial cells from oxidative stress-induced apoptosis by inactivating 4-HNE and thereby precluding mitochondrial membrane damage. Future *in vivo* studies are required to show that 4-HNE pretreatment attenuates hyperoxia-induced endothelial cells death and increased lung permeability.

In summary, these findings suggest that lung vascular endothelial cells can be protected against hyperoxic damage through ALDH2 activation by use of Alda-1. Administration of Alda-1 has the potential to be applied as a novel therapeutic tool to preclude and/or diminish oxygen therapy-associated lung injury in patients with ALI/ARDS. Further *in vivo* studies are needed to determine the efficacy and tolerability of Alda-1 treatment to assess its clinical usefulness.

## MATERIALS AND METHODS

### Cell culture

HMVEC were purchased from Lonza and maintained in EGM-2 MV bullet kit prepared according to manufacturer instructions (Lonza, Walkersville, MD). Cells were cultured at 37 ºC in a 5% carbon dioxide humidified incubator to maintain sufficient cell growth. The cultured cells were validated for confluence (around 70%) and exposed to hyperoxia for 48 hours with and without Alda-1 pretreatment.

### Alda-1

Alda-1 (N-(1,3-Benzodioxol-5-ylmethyl)-2,6-dichlorobenzamide) (St. Louis, MO) was purchased from Sigma Aldrich, protected from light, and 20µM concentrations were used for experiments.

### Oxidative stress assay

Oxidative stress was measured by OxiSelect™ 4-HNE adduct competitive ELISA kit (Cell Biolabs, San Diego, CA) according to the instructions. Whole cell lysate (50 µg protein equivalent) for each sample was used for the assay.

### Western blot

HMVEC were cultured, confluence was confirmed (70%), and the cells were exposed to hyperoxia with or without Alda-1 for 48 hours. The oxygen level was monitored by the proOX p100 sensor (Biospherix, NY). After hyperoxia, the cells were harvested and the collected cell pellets were suspended in lysis buffer (20mM Tris HCL, pH7.4, 150mM Nacl, 0.5% Triton-x 100). After centrifugation at high speed (14000g) for 15 minutes at 4° C, the supernatant was collected. The protein concentration of each sample was determined by BCA assay kit (Pierce, Rockford, IL). Equivalent amounts of protein (20µg) were run using SDS-PAGE on a 4-20% tris-glycine gel (BioRad, Hercules, CA). The protein samples on the gel were then transferred to PVDF membranes. After blocking in 5% skim milk, the membranes were incubated with primary and then secondary antibodies. The antibodies used are anti-Cytochrome C, anti β-actin (Cell signaling technologies, Beverly, MA), anti-Bax (Santa Cruz, Dallas, TX), anti-ALDH2 (Novus Bio, Centennial, CO). The secondary antibodies used were HRP-conjugated anti-rabbit and mouse IgG antibodies (Jackson Immunoresearch laboratory, Inc., Westgrove, PA). The bands were developed using pierce ECL (Thermo Fischer scientific, Hudson, NH) and the detected bands were scanned and analyzed densitometrically by NIH ImageJ software.

### JC-1 staining

HMVEC were plated at a density of 10^4^ cells in CELL view dish with glass bottom (Thomas scientific, Swedesboro, NJ). When cells reached 70% confluence, they were exposed to hyperoxia for 48 hours with or without Alda-1. Following hyperoxia, the HMVEC-L were washed by HBSS solution (with CaCl_2_ and MgCl_2_) (Gibco, Waltham, MA). After washes, the HMVEC-L were treated with JC-1 (2.5 µM) (Thermo Fisher, Waltham, MA) at 37° C for 15 minutes and washed in HBSS three times and placed into the media. Live cell imaging was conducted using fluorescence microscopy (Olympus, Tokyo) and magnification was 200X. Around 100 cells were quantified from each group. The green and red images were captured in fluorescence and the mean signal intensity of each image was quantified using ImageJ software.

### ALDH2 activity

The enzymatic activity of ALDH2 was measured by transformation of acetaldehyde to acetic acid [16]. At the end of each treatment, HMVEC-L were harvested and the cell pellets were re-suspended in 300 µL of ALDH2 assay buffer (10 mM DTT, 100 mM Tris-HCl pH 8.0, 20% glycerol, 1% Triton X-100). Cell suspension was then centrifuged at 55000g for 30 minutes at 4° C. The supernatant was collected and utilized to detect the activity of ALDH2. The assay mixture (1 mL) contains 10mM NAD+, 100mM sodium pyrophosphate, 10 mM acetaldehyde, and 100 µg protein equivalent. Acetaldehyde was added to the cuvette immediately before the reaction was started. The NADH generated de novo was determined by spectrophotometric absorbance at 340 nm. The activity of the ALDH2 enzyme was expressed as nmol NADH/ minute/ mg protein.

### Statistical Analysis

All experiments were conducted with n=3 per group and values were indicated as means ± SE. Statistical significance was calculated by using Microsoft excel and T-tests were two tailed; values less than p<0.05 were considered significant (*p<0.05, **<0.005).
